# A blood-brain barrier-penetrant AAV gene therapy improves neurological function in symptomatic mucolipidosis IV mice

**DOI:** 10.1016/j.omtm.2024.101269

**Published:** 2024-05-21

**Authors:** Madison L. Sangster, Martha M. Bishop, Yizheng Yao, Jessica F. Feitor, Sanjid Shahriar, Maxwell E. Miller, Anil K. Chekuri, Bogdan Budnik, Fengfeng Bei, Yulia Grishchuk

**Affiliations:** 1 Center for Genomic Medicine and Department of Neurology, Massachusetts General Hospital Research Institute and Harvard Medical School, 185 Cambridge St, Boston, MA 02114, USA; 2 Department of Neurosurgery, Brigham and Women’s Hospital, Harvard Medical School, 75 Francis St, Boston, MA 02115, USA; 3 Wyss Institute for Biologically Inspired Engineering, Harvard University, 201 Brookline Avenue, Boston, MA 02215, USA; 4 Grousbeck Gene Therapy Center, Schepens Eye Research Institute, Massachusetts Eye and Ear Infirmary, Harvard Medical School, 20 Staniford St, Boston, MA 02114, USA

**Keywords:** TRPML1, lysosomal disorder, AAV, adeno-associated vector, gene transfer, pediatric neurological disease, mouse model

## Abstract

Mucolipidosis IV (MLIV) is a rare, autosomal recessive, lysosomal disease characterized by intellectual disability, motor deficits, and progressive vision loss. Using adeno-associated vector 9 (AAV9) and AAV-PHP.B as delivery vectors, we previously demonstrated the feasibility of modifying disease course in a mouse model of MLIV by the human *MCOLN1* gene transfer. Here, using a primate-enabling capsid AAV.CPP.16 (CPP16), we constructed a new, clinic-oriented *MCOLN1* gene expression vector and demonstrated its efficacy in the preclinical model of MLIV. Systemic administration of CPP16-MCOLN1 in adult symptomatic *Mcoln1*^*−/−*^ mice at a dose of 1e12 vg per mouse resulted in *MCOLN1* expression in the brain and peripheral tissues, alleviated brain pathology, rescued neuromotor function, and completely prevented paralysis. Notable expression of *MCOLN1* transcripts was also detected in the retina of the mouse, which had exhibited significant degeneration at the time of the treatment. However, no increase in retinal thickness was observed after gene therapy treatment. Our results suggest a new AAV-based systemic gene replacement therapy for the treatment of MLIV that could be translated into clinical studies.

## Graphical abstract

**Figure:**
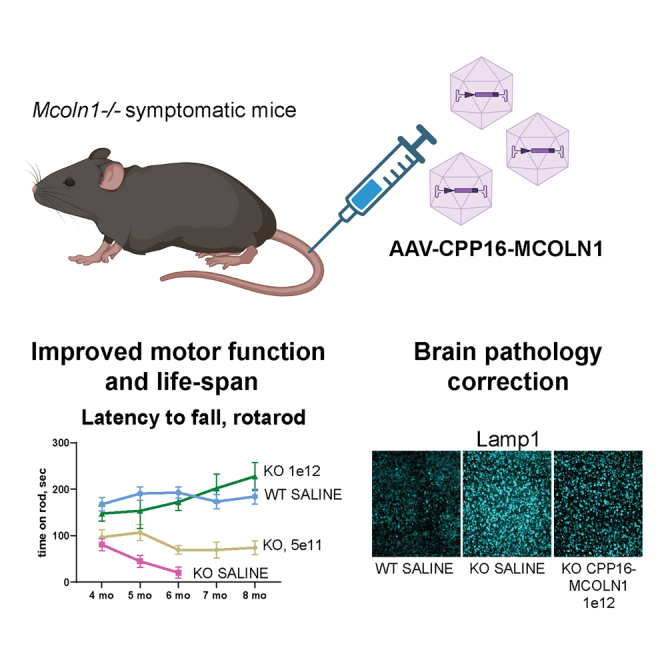

## Teaser

MLIV is a rare neurological disease of childhood with extremely high unmet need. Here, Grishchuk and colleagues show that AAV-CPP16-mediated gene transfer of the MLIV causative *MCOLN1* gene to symptomatic MLIV mice restores neurofunction, delays paralysis, and alleviates brain pathology, indicating a promising therapeutic approach for clinical development.

## Introduction

Mucolipidosis IV (MLIV) is a rare pediatric neurological disease caused by loss-of-function mutations in the *MCOLN1* gene.^1^ MLIV was first described in 1974,^2^ and the causative gene was identified in 1999.^3^^,^^4^^,^^5^ Patients typically present with corneal clouding and delayed developmental milestones in the first year of life and reach a plateau in psychomotor development by 2 years of age. Early onset of axial hypotonia and signs of pyramidal and extrapyramidal motor dysfunction prevent independent ambulation in the majority of MLIV patients and severely limit fine motor function. Although MLIV was originally described as a static neurodevelopmental disorder, progressive neurological deterioration has recently been documented during the second decade of life.^6^ In congruence with the clinical course, brain imaging has demonstrated stable white matter abnormalities (corpus callosum hypoplasia and dysgenesis, and white matter lesions) with the emergence of subcortical volume loss and cerebellar atrophy in older patients.^7^^,^^8^ Visual impairment is also a prominent feature of MLIV, with progressive retinal dystrophy and optic nerve atrophy leading to blindness by the second decade of life,^8^^,^^9^^,^^10^^,^^11^ further impeding function and negatively impacting quality of life. At present, the standard of care for MLIV primarily focuses on symptom management, and no disease-modifying treatments are available.

*MCOLN1* encodes the late endosomal/lysosomal non-selective cation channel TRPML1, which regulates lysosomal ion balance and is directly involved in multiple lysosome-related pathways, including Ca^2+^-mediated fusion/fission with the lysosomal membrane, mammalian target of rapamycin signaling, TFEB activation, lysosomal biogenesis,^12^^,^^13^^,^^14^^,^^15^ and autophagosome formation.^16^ Additionally, its role in Fe^2+^ transport and regulating brain iron homeostasis has also been demonstrated.^17^^,^^18^

Important insights into the pathophysiology of the disease have been obtained using the *Mcoln1* knock-out mouse model we developed.^19^^,^^20^^,^^21^^,^^22^ *Mcoln1*^*−/−*^ mice recapitulate the clinical and pathological phenotype of MLIV patients, including motor deficits, retinal degeneration, corpus callosum hypoplasia, microgliosis, astrocytosis, and, later in the disease, partial loss of Purkinje cells. The first signs of motor dysfunction in *Mcoln1*^*−/−*^ mice appear at the age of 2 months in the form of reduced vertical activity in the open field test.^18^^,^^23^ Later in life, motor deficits present as clasping starting from around 3 months, shorter retention on the accelerating rotarod starting from 4 months, gait deficits noticeable at around 6 months of age, and progress to complete hindlimb paralysis by 6–7 months, at which point *Mcoln1*^*−/−*^ mice are being humanely euthanized.^23^

Recently, we showed that systemic *MCOLN1* gene transfer using adeno-associated vector (AAV)-PHP.B, a mouse-restricted blood-brain barrier (BBB)-penetrant capsid,^24^ can fully reverse neurological dysfunction in the MLIV mouse model when administered to symptomatic 2-month-old *Mcoln1*^*−/−*^ mice, demonstrating the feasibility of altering the disease course of MLIV after symptom onset using gene therapy.^23^ Additionally, intracerebroventricular (ICV) injection of a self-complementary, *MCOLN1*-expressing AAV9 vector (scAAV9-MCOLN1) in neonatal *Mcoln1*^*−/−*^ pups was also efficacious, as wide brain transduction was achieved using this route of administration in young animals. However, when scAAV9-MCOLN1 was intravenously administered in adult symptomatic mice leading to successful overexpression of the transgene in the periphery but little transduction in the brain, no therapeutic effect was observed.^23^ These studies suggest that broad brain targeting by a human-translatable vector is essential in achieving optimal therapeutic outcomes when developing AAV gene therapy for MLIV. Despite their value in demonstrating proof of concept, the clinical translatability of these findings was limited due to species-restricted BBB penetration properties of AAV-PHP.b and a non-translatable neonatal delivery method of scAAV9. To overcome these limitations, in this study, we tested the preclinical efficacy of human *MCOLN1* gene transfer using a recently reported new AAV capsid, AAV.CPP.16 (CPP16). Compared with its parent capsid AAV9, systemic administration of CPP16 demonstrates a more than 5-fold enhancement in transduction of the CNS in both mice and non-human primates.^25^ Although it may not be the most potent in overcoming the mouse BBB as compared with other recently developed capsids,^26^^,^^27^ the translatability of CPP16 from rodents to primates prompted us to examine whether systemic delivery of CPP16-MCOLN1 would restore sufficient *MCOLN1* expression in the brain and yield a meaningful functional outcome. The intravenous route of administration was selected over intra-cerebrospinal fluid delivery to achieve delivery of the vector to as many neurons throughout the brain as possible, including in the deep brain regions.^25^^,^^28^ We found that intravenous delivery of this vector in symptomatic *Mcoln1*^*−/−*^ mice led to a dose-dependent improvement of motor function, significantly delayed time to paralysis, and corrected brain pathology in treated *Mcoln1*^*−/−*^ animals. These data suggest that AAV.CPP.16-mediated systemic gene replacement therapy could be a promising approach for treating patients with MLIV.

## Results

### Systemic administration of CPP16-MCOLN1 in young adult symptomatic *Mcoln1*^*−/−*^ mice resulted in dose-dependent restoration of neuromotor function and delayed onset of paralysis

**Figure 1:**
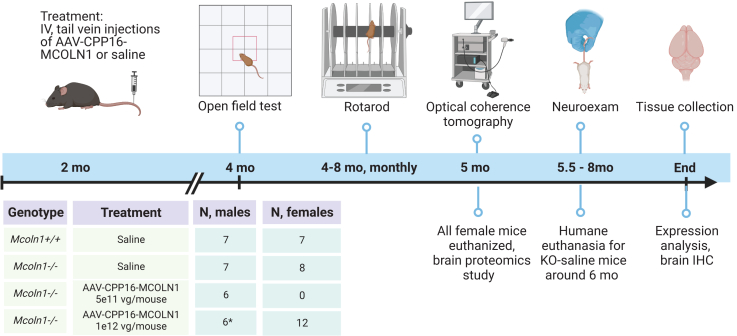
Schematic representation of CPP16-MCOLN1 efficacy study in *Mcoln1*^*−/−*^ mice and study groups Note that male and female *Mcoln1*^*−/−*^ mice have very similar disease phenotype. In this study, we used male and female cohorts for assessment of different outcomes past 4 months of age to allow collection of the complementary outcomes instead of duplicating assessments in male and female cohorts to evaluate sex-specific differences. ∗One male developed severe health concern (penile prolapse) and had to be euthanized at 3 months of age. Assessment of efficacy after 3 months of age is reported for five mice in *Mcoln1*^*−/−*^- CPP16-MCOLN1, 1e12 group.

**Figure 2:**
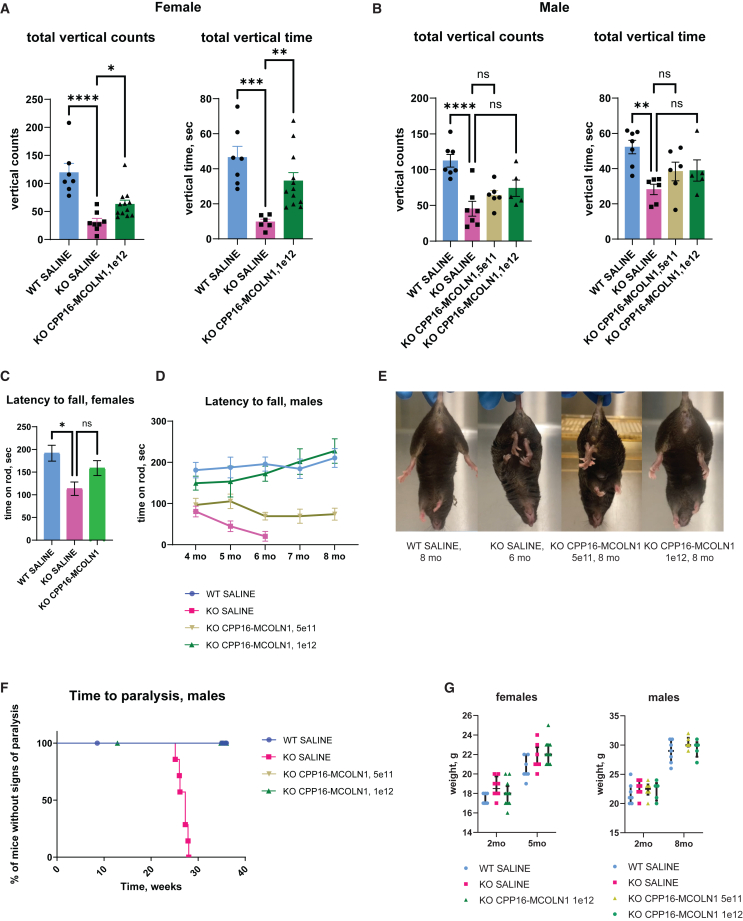
Delayed paralysis and dose-dependent restoration of neurological function after intravenous administration of CPP16-MCOLN1 in symptomatic *Mcoln1*^*−/−*^ mice (A and B) Measurements of the vertical activity in the open field test, represented as total vertical counts and total vertical time in the open field arena, in female (A) and male (B) mice show significantly decreased activity in the 4 months old *Mcoln1*^*−/−*^ (knockout [KO]) mice treated with saline compared with saline-treated WT controls, and significant recovery in the *Mcoln1*^*−/−*^ mice that were treated with CPP16-MCOLN1 at the symptomatic stage of the disease at 2 months of age. Data presented as individual data points per animal, mean values, and SEM; two *Mcoln1*^*−/−*^-saline female and one *Mcoln1*^*−/−*^-saline male mice were identified as outliers using ROUT (Q = 1%) and Grubbs (alpha = 0.05) methods and excluded from stat analysis; group comparisons made using one-way ANOVA test; represented p values were corrected for multiple comparisons between individual groups. (C) Rotarod performance in female mice at 4 months of age. n (WT SALINE) = 7; n (*Mcoln1*^*−/−*^ SALINE) = 8; n (*Mcoln1*^*−/−*^ CPP16-MCOLN1, 1e12) = 12; one-way ANOVA test p = 0.0199; Dunnet’s multiple comparison test p (WT SALINE vs. *Mcoln1*^*−/−*^ SALINE) = 0.0114; p (*Mcoln1*^*−/−*^ SALINE vs. *Mcoln1*^*−/−*^ CPP16-MCOLN1). (D) Dose-dependent long-term improvement of the rotarod performance presented as average latency to fall indicates better motor function, balance and coordination in the *Mcoln1*^*−/−*^ mice treated with CPP16-MCOLN1. The data shown as mean and SEM, n (WT SALINE) = 7; n (*Mcoln1*^*−/−*^ SALINE) = 7; n (*Mcoln1*^*−/−*^ CPP16-MCOLN1, 5e11) = 6; n (*Mcoln1*^*−/−*^ CPP16-MCOLN1, 1e12) = 5 (note that one of the six mice in this group developed penile prolapse at 3 months and was euthanized). The same number of animals in each group was used for each time point except only four of the seven *Mcoln1*^*−/−*^ SALINE mice remained available for testing at 6 month of age, as three mice had been euthanized die to onset of hindlimb paralysis. p < 0.0001 (one-way ANOVA); Dunnet’s multiple comparisons: p (WT SALINE vs. *Mcoln1*^*−/−*^ SALINE) < 0.0001; p (*Mcoln1*^*−/−*^ SALINE vs. *Mcoln1*^*−/−*^ CPP16-MCOLN1, 5e11) = 0.152; p (*Mcoln1*^*−/−*^ SALINE vs. *Mcoln1*^*−/−*^ CPP16-MCOLN1, 1e12) <0.0001. (E) Representative images of mice in all treatment/genotype groups showing rescue of clasping in the *Mcoln1*^*−/−*^ mice treated with 1e12 vg/mouse, but not with 5e11 vg/mouse of CPP16-MCOLN1. (F) Systemic administration of CPP16-MCOLN1 at 2 months of age significantly delays time to paralysis in *Mcoln1*^*−/−*^ male mice. The criterion for paralysis was loss of righting reflex when mouse failed to rotate itself in upright position after placing on a side within 10 s; log rank test p value is less than 0.0001. (G) No significant weight changes have been observed in mice treated with CPP16-MCOLN1. Data presented as median values and interquartile range.

The self-complimentary CPP16 vector for this study was produced using the same *MCOLN1* expression construct that we previously created to package the scAAV9-MCOLN1 vector, which showed efficacy in *Mcoln1*^*−/−*^ mice when delivered via neonatal ICV administration.^23^ In this vector, the expression of human *MCOLN1* cDNA is driven by a short ubiquitous synthetic promoter JeT.^23^ To test the efficacy of scAAV-CPP16-MCOLN1 (later in text referred to as CPP16-MCOLN1), cohorts of male and female *Mcoln1*^*−/−*^ and wild-type (WT) littermate control mice received tail-vein injections of either 5e11 vg, 1e12 vg CPP16-MCOLN1, or saline at the age of 2 months (see Figure 1 for details, including animal numbers), when the *Mcoln1*^*−/−*^ mice develop decline of motor function in the form of vertical activity. Sex matching was not performed as no gender-specific disease manifestations were observed in human MLIV patients or in our mouse model.^23^ Male animals were selected for long-term behavioral monitoring because of their better consistency in performing. Efficacy was assessed using our established standard outcome measures, including open field and rotarod tests, and assessment of clasping and righting reflexes. The experimental design, including group size and order of testing, is shown in Figure 1. Reduction of vertical activity is one of the first signs of motor function decline in *Mcoln1*^*−/−*^ mice that can be measured in the open field test starting at 2 months of age.^22^^,^^23^ Consistent with our previous findings, a significant reduction of vertical movements and time spent in the vertical position was observed in saline-treated *Mcoln1*^*−/−*^ mice compared with their WT littermates in both sexes at the age of 4 months (Figures 2A and 2B). In females, intravenous administration of CPP16-MCOLN1 at 1e12 vg/animal led to significant rescue of vertical movements and vertical time, indicating the restoration of neurological function (Figure 2A). In males, we observed a similar trend toward higher vertical activity in *Mcoln1*^*−/−*^ animals treated with either 5e11 or 1e12 vg/mouse of CPP16-MCOLN1 as compared with the saline-treated *Mcoln1*^*−/−*^ group. Statistical significance between saline- and CPP16-MCOLN1-treated *Mcoln1*^*−/−*^ males was not detected, likely due to the small sample size and inclusion of the additional, lower dose group in the analysis.

We next evaluated motor function, balance, and coordination using the rotarod test. Female mice were tested once at 4 months of age before tissue collection at 5 months of age (Figure 2C). Saline-treated *Mcoln1*^*−/−*^ females fall off the rotating rod sooner than saline-treated healthy controls. The CPP16-MCOLN1-treated *Mcoln1*^*−/−*^ female group showed a trend toward higher rod retention in this test. However, the difference was not statistically significant using a one-way ANOVA test for multiple comparisons.

The male cohort of mice was used for continuous monthly rotarod testing starting at 4 months of age. Compared with healthy littermates, saline-treated *Mcoln1*^*−/−*^ mice showed a lower average latency to fall starting at the age of 4 months (Figure 2D). Gradually, saline-treated *Mcoln1*^*−/−*^ mice developed hindlimb weakness and were eventually euthanized at around 6 months of age due to hindlimb paralysis. Importantly, *Mcoln1*^*−/−*^ mice that received 1e12 vg of CPP16-MCOLN1 (high dose) showed comparable performance on rotarod with the control healthy littermates up to the end of the trial at 8 months of age. Furthermore, no hindlimb clasping was observed in these animals, and their overall appearance was indistinguishable from that of saline-treated WT mice (Figure 2E). *Mcoln1*^*−/−*^ group treated with 5e11vg of CPP16-MCOLN1 per animal (low dose) did not perform as well on rotarod as the animals in the high-dose group, showing lower rod retention time (Figure 2D). The low-dose group also showed no rescue of clasping (Figure 2E) and had a scruffy coat appearance resembling saline-treated *Mcoln1*^*−/−*^ mice. Interestingly, despite the general ill appearance of the *Mcoln1* knock-out mice treated with the low dose of CPP16-MCOLN1, none of these mice developed signs of hind-limbs paralysis throughout the 8-month study (Figure 2F). The untreated or saline-treated *Mcoln1*^*−/−*^ mice develop signs of paralysis that warrant euthanasia at around 6 months of age. Therefore, we used the time-to-paralysis measure as a surrogate of lifespan in this mouse model. None of the CPP16-MCOLN1 treated *Mcoln1*^*−/−*^ mice showed any sign of paralysis by 8 months of age and outlived the saline-treated group by at least 2 months, demonstrating significantly delayed time to paralysis and improved lifespan. We observed no weight changes or health concerns in the CPP16-MCOLN1-treated mice (Figure 2G). In conclusion, our data show a dose-dependent response of CPP16-MCOLN1 gene therapy in MLIV with neuromotor functions fully rescued by a single, high-dose systemic administration.

### Expression of *MCOLN1* transgene in the CNS and peripheral tissues

**Figure 3:**
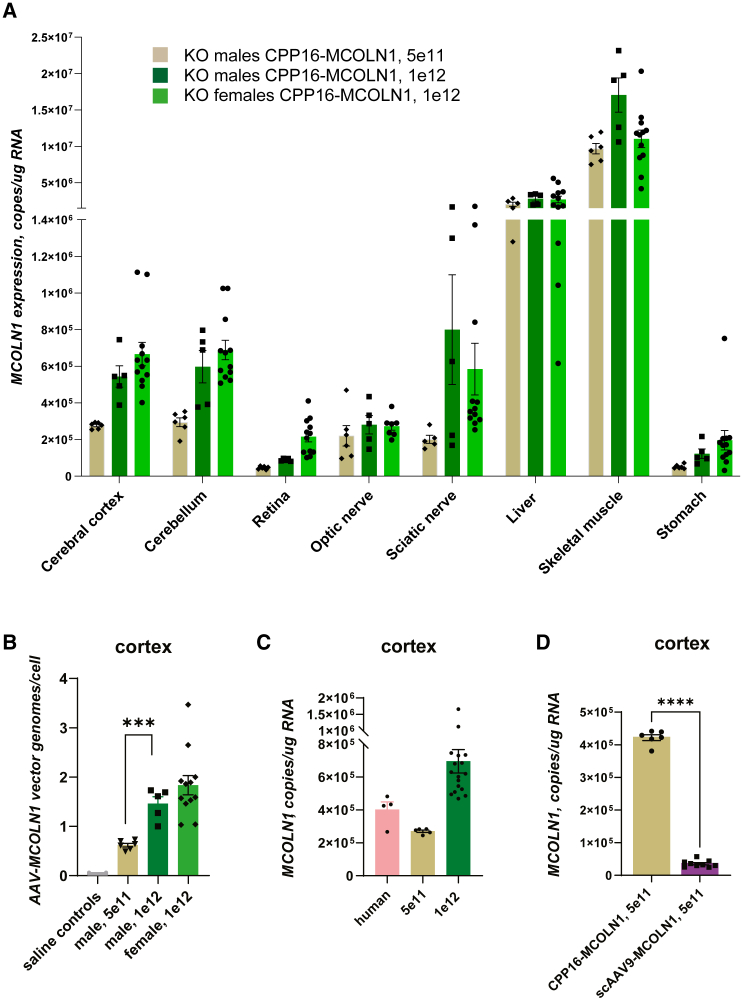
*MCOLN1* expression analysis in postmortem mouse tissues shows transduction in CNS and peripheral tissues (A) qRT-PCR analysis of the *MCOLN1* transcripts showing transgene overexpression in the cerebral cortex, cerebellum, retina, optic nerve, sciatic nerve, liver, quad muscle, and stomach. Data show individual, mean values and SEM. Unless specifically noted, all samples from CPP16-MCOLN1-treated male and female *Mcoln1*^*−/−*^ groups were included in this assay, i.e., n (*Mcoln1*^*−/−*^ CPP16-MCOLN1, 1e12, female) = 12; n (*Mcoln1*^*−/−*^ CPP16-MCOLN1, 1e12, male) = 5 (note that one of the six mice in this group developed penile prolapse at 3 months and was excluded from analysis); n (*Mcoln1*^*−/−*^ CPP16-MCOLN1, 5e11, male) = 6. Only 7 samples were included in optic nerve dataset in the female *Mcoln1*^*−/−*^ CPP16-MCOLN1, 1e12 group, and five samples were included in the liver and sciatic nerve datasets in the male *Mcoln1*^*−/−*^ CPP16-MCOLN1, 5e11 group due to technical errors or unavailable RNA. (B) qPCR vector genome copy analysis showing CPP16-MCOLN1 vector biodistribution in the cerebral cortex after intravenous administration to 2-month-old mice. (C) qRT-PCR analysis showing AAV-CPP16-driven human *MCOLN1* expression in the mouse cortex vs. endogenous *MCOLN1* expression in the human cortex. p (one-way ANOVA) = 0.0048. Five of the six samples were included in the male *Mcoln1*^*−/−*^ CPP16-MCOLN1, 5e11 group due to unavailable RNA. (D) qRT-PCR analysis in *Mcoln1*^*−/−*^ mice injected intravenously with 5e11vg/animal of either self-complimentary CPP16-MCOLN1 or AAV9-MCOLN1 shows that CPP16 is superior in transducing the brain from systemic flow as compared with scAAV9. Data presented as individual data points, mean values, and SEM. p, unpaired t test. ∗∗∗p < 0.001; ∗∗∗∗p < 0.0001.

qRT-PCR analysis of the *MCOLN1* transgene expression in postmortem tissues showed a dose-dependent increase of mRNA transcripts in the brain regions (cortex and cerebellum), retina, sciatic nerve, skeletal muscle, and stomach of *Mcoln1*^*−/−*^ males treated with 5e11 or 1e12 vg/mouse of CPP16-MCOLN1 (Figure 3A). No significant differences were detected between male and female groups treated with CPP16-MCOLN1 at 1e12 vg/mouse, except in the retina, where higher expression was detected in female mice. Notably, transduction and transgene expression in the retina with systemic administration of CPP16 capsid have not been reported previously. In line with the previously published data on CPP16 biodistribution in mouse tissues,^25^ we observed high expression of *MCOLN1* transcripts in the liver. Remarkably high expression was also detected in the skeletal muscle.

Vector genome quantification analysis in the cerebral cortex showed a dose-dependent increase of viral genome copies per cell in the male groups from the mean of 0.61 (±0.037, SEM) in the 5e11 vg/mouse group to 1.46 (±0.14) in the 1e12 vg/mouse group (Figure 3B). An average of 1.83 (±0.195) vg/cell was detected in the female mice treated with 1e12 vg of the vector. These data, together with the motor function outcomes described earlier, suggest that average cortical biodistribution higher than 0.61 vg/cell may be required to obtain restoration of the neurological function in MLIV.

To assess how therapeutic levels of CPP16-mediated *MCOLN1* expression determined in the mouse tissue are compared with the amount of endogenous human *MCOLN1* expression, we measured *MCOLN1* mRNA in four human cortical samples (young adult male; Maryland Brain Biobank) and compared them with the human *MCOLN1* transgene mRNA levels in the cortices of the *Mcoln1*^*−/−*^ mice treated with CPP16-MCOLN1 (Figure 3C). We found that treatment with the low dose of CPP16-MCOLN1 (5e11 vg per mouse) resulted in the expression of *MCOLN1* mRNA lower than the endogenous level in the human cortex, while treatment of the high dose (1e12 vg per mouse) led to, on average, a higher than endogenous level *MCOLN1* transcript expression.

The superiority of CPP16’s ability to transduce brain tissue from systemic flow over AAV9 has previously been demonstrated.^25^ To confirm this in our study, we performed a direct comparison of *MCOLN1* mRNA levels in the cerebral cortex tissue of *Mcoln1*^*−/−*^ mice that received either self-complimentary AAV9 or self-complimentary CPP16 vectors carrying the same expression cassette via the same route of administration (tail vein injection). Consistent with the previous report, we found that CPP16-mediated *MCOLN1* expression was 12-fold higher than that achieved by AAV9 (Figure 3D).

### Systemic treatment with CPP16-MCOLN1 did not alleviate retinal pathology

Eye pathology in *Mcoln1*^*−/−*^ mice includes thinning of the photoreceptor layer, reduced levels of rhodopsin, and significantly decreased dark-adapted a- and b-response, indicative of the rod cells impairment.^21^ Remarkably, this retinal phenotype in *Mcoln1*^*−/−*^ mice was found to be present early, at 1 month of age, and was static, in contrast with progressive retinal, corneal, and optic nerve pathology in MLIV patients that leads to complete blindness in the second decade of life. Since our expression data showed expression of the human *MCOLN1* transgene in the *Mcoln1*^*−/−*^ mouse retina after intravenous administration of CPP16-MCOLN1, we set out to assess whether this expression resulted in the correction of retinal thinning. Retinal thickness was measured in live mice using high-definition spectral domain optical coherence tomography (SD-OCT). Male 5-month-old WT and *Mcoln1*^*−/−*^ mice that received either saline or 1e12 vg of CPP16-MCOLN1 were used. The SD-OCT showed significant retinal thinning, with a reduced thickness of the photoreceptor (outer nuclear) layer and the outer photoreceptor segments in saline-treated *Mcoln1*^*−/−*^ mice compared with saline-treated WT, confirming retinal degeneration. Unfortunately, no improvement of the retinal or outer nuclear layers was detected in the *Mcoln1*^*−/−*^ mice treated with CPP16-MCOLN1 (Figure S1). Given that retinal phenotype is fully developed in the *Mcoln1*^*−/−*^ mice by 1 month of age and is not progressive later in life, and CPP16-MCOLN1 in our study was administered when mice reached 2 months of age, the lack of therapeutic benefit may be a result of belated intervention.

### CPP16-MCOLN1 reduces brain pathology in *Mcoln1*^*−/−*^ mice

**Figure 4:**
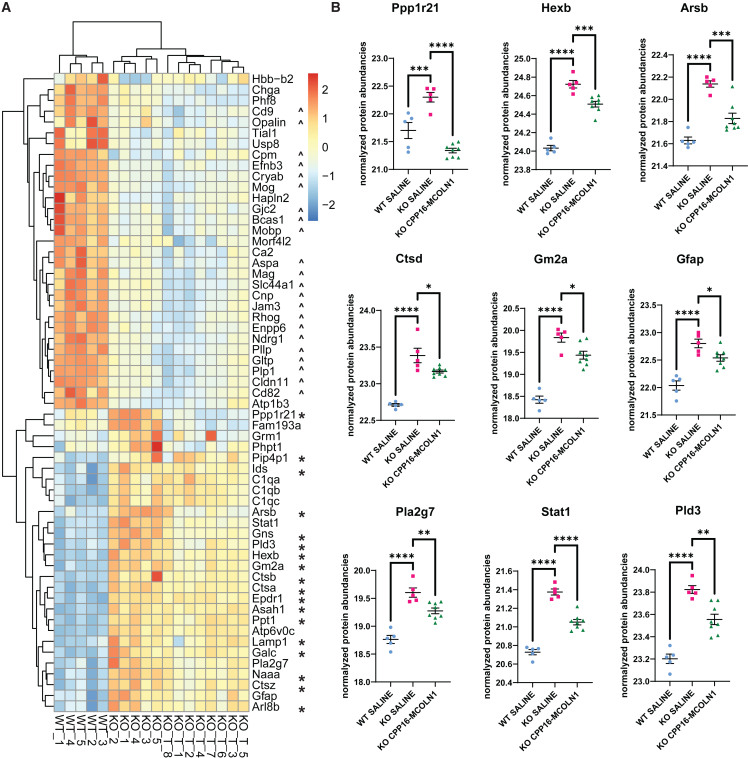
Intravenous administration of CPP16-MCOLN1 in symptomatic *Mcoln1*^*−/−*^ mice partially corrects the upregulation of lysosomal proteins in the brain (A) Heatmap showing downregulated and upregulated proteins (log10 *p* < 0.05, log2FC > 0.5) in *Mcoln1*^*−/−*^-saline cortical homogenates from 5-month-old female mice compared with WT-saline littermates and corresponding protein abundancies in *Mcoln1*^*−/−*^ CPP16-MCOLN1-treated mice. ∗Lysosomal proteins; ˆProteins enriched in oligodendrocyte cell lineage and myelin. (B) Individual proteins in endosomal/lysosomal compartment (top) or glial and immune-related proteins, demonstrating a significant increase in saline-treated knockout (KO) and their correction in KO mice treated with CPP16-MCOLN1. p, one-way ANOVA and Dunnett test for multiple comparisons, ∗p < 0.05, ∗∗p < 0.01, ∗∗∗p < 0.001, ∗∗∗∗p < 0.0001. See also Figures S2, S3, Tables S1, S2, S3, and S4.

An increased abundance of the lysosomal proteins and decreased levels of oligodendrocyte and myelin-related proteins are molecular hallmarks in the brain of symptomatic *Mcoln1*^*−/−*^ mice.^29^ To assess if administration of CPP16*-*MCOLN1 rescued brain pathology in *Mcoln1*^*−/−*^ mice, we next performed LC-MS/MS proteomics analysis using cortical tissues of 5-month-old female *Mcoln1*^*−/−*^ mice as well as WT control mice. In accordance with previous findings, we observed a remarkably similar broad upregulation of the lysosomal proteins and downregulation of myelination and oligodendrocyte protein signature in the *Mcoln1*^*−/−*^ cortex (Figure 4A). In total, 3,490 proteins were identified (Table S1). Mouse albumin and keratin entries and non-mouse proteins were removed from these datasets (the list of all typical contaminants is available in Table S2). Principal component analysis (PCA) separated *Mcoln1*^*−/−*^ saline, *Mcoln1*^*−/−*^ CPP16-MCOLN1, and WT saline samples (Figure S2). We detected 28 upregulated and 31 downregulated proteins in the cortical tissue of saline-treated *Mcoln1*^*−/−*^ mice as compared with WT-saline group, with log2 fold change cutoff at 0.5 (fold change of ±1.4) and log10 p value cut off at 1.3 (p < 0.05) (Figures 4A and S3; Table S3). Eighteen of the 28 upregulated proteins were lysosomal. Remarkably, we observed a broad reduction of lysosomal protein levels in the *Mcoln1*^*−/−*^ mice treated with CPP16-MCOLN1. Selected examples including Protein phosphatase 1 regulatory subunit 21 (Ppp1r21), beta-hexosaminidase subunit B, arylsulfatase B, cathepsin D, and ganglioside GM2 activator are presented in Figure 4B. In addition, we observed a reduction of the astrocytosis marker Glial fibrillary acidic protein (Gfap) and inflammation-linked proteins including Platelet-activating factor acetylhydrolase, signal transducer and activator of transcription 1, 5′-3′ exonuclease PLD3 in CPP16-MCOLN1-treated samples. These data demonstrate at least partial correction of lysosomal and pro-inflammatory phenotype in the *Mcoln1*^*−/−*^ mouse brain after gene transfer of human *MCOLN1*. Our liquid chromatography tandem mass spectrometry (LC-MS/MS) data showed no recovery of myelin or oligodendrocyte-related proteins in *Mcoln1*^*−/−*^ mice treated with CPP16-MCOLN1. The complete proteomic dataset is presented in Table S4.

**Figure 5:**
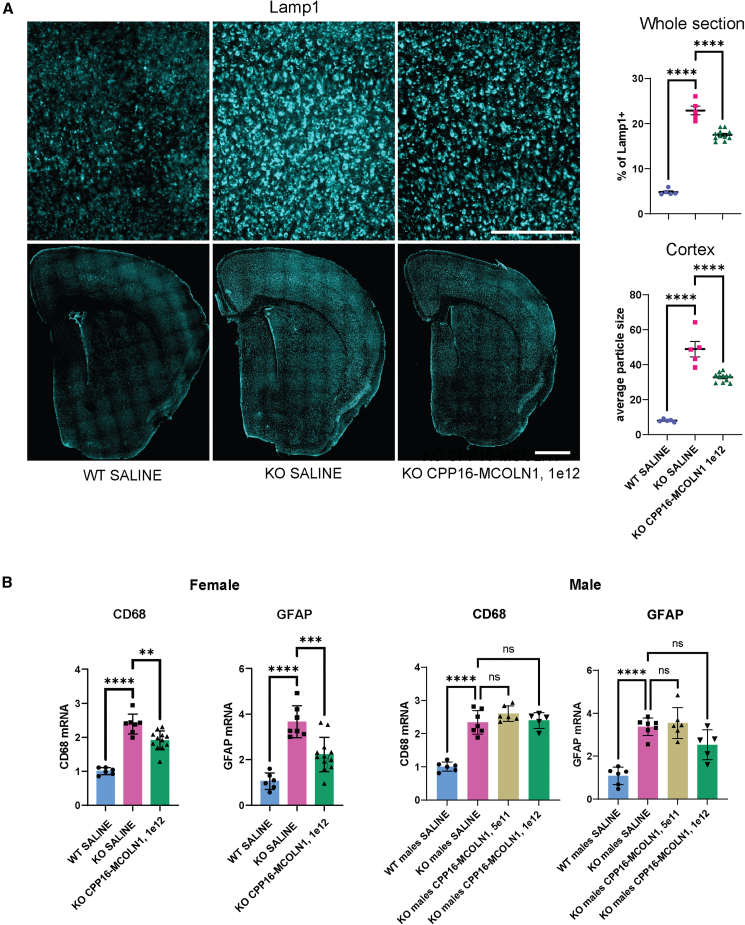
Intravenous administration of CPP16-MCOLN1 in symptomatic *Mcoln1*^*−/−*^ mice reduces the accumulation of Lamp1-positive lysosomal aggregates and reduces activation of astrocytosis and microgliosis in *Mcoln1*^*−/−*^ mouse brain (A) Representative images of Lamp1 staining in the mouse brain at higher (top; scale bar, 0.25 mm) and lower (bottom; scale bar, 1 mm) magnification and image quantification showing average Lamp1^+^ particle size and % of Lamp1^+^ staining area in the coronal whole hemisphere sections. Data show individual, mean values and SEM, n (WT-saline, female) = 5; n (*Mcoln1*^*−/−*^saline, female) = 5; n (*Mcoln1*^*−/−*^ CPP16-MCOLN1, 1e12, female) = 11. Comparisons were done using ordinary one-way ANOVA with Dunnett’s test for multiple comparisons using GraphPad Prism v.9; ∗∗p < 0.01, ∗∗∗∗p < 0.0001. (B) qRT-PCR analysis of the microglia activation marker *Cd68* and astrocytosis marker *Gfap* showing significant upregulation of the corresponding transcripts in the cerebral cortex of *Mcoln1*^*−/−*^ mice treated with saline compared with saline-treated WT littermate mice and significant reduction of the *Cd68* and *Gfap* transcripts in the *Mcoln1*^*−/−*^ female mice treated with CPP16-MCOLN1, while no significant differences were observed between saline and CPP-treated *Mcoln1*^*−/−*^ males. Data show individual, mean values and SEM, n (WT-saline, female) = 6; n (*Mcoln1*^*−/−*^saline, female) = 7; n (*Mcoln1*^*−/−*^ CPP16-MCOLN1, 1e12, female) = 11. Comparisons were done using ordinary one-way ANOVA with Dunnett’s test for multiple comparisons using GraphPad Prism v.9; ∗∗p < 0.01, ∗∗∗p < 0.001, ∗∗∗∗p < 0.0001.

To confirm our observation of lysosomal protein correction using LC-MS/MS proteomics, we performed immunohistochemistry to examine lysosomal pathology. Lamp1 is broadly used as a lysosomal marker, and increases in the percentage of Lamp1^+^ staining and size of Lamp1^+^ particles are indicative of lysosomal abnormality in *Mcoln1*^*−/−*^ mice.^23^ We found that intravenous administration of the CPP16-MCOLN1 vector led to a significant reduction of both % of the stained area and particle size measurements (Figure 5A), demonstrating a reduction of the lysosomal pathology in the treated *Mcoln1*^*−/−*^ group.

We further performed qRT-PCR analysis to examine astrocytosis and microgliosis, which are early hallmarks of the MLIV brain pathology reported in the human tissue and the MLIV mouse model.^22^^,^^30^^,^^31^^,^^32^ As expected, significant increases in mRNA transcripts of the astrocytosis marker *Gfap* and microgliosis marker *Cd68* were observed in the cortical tissue of saline-treated *Mcoln1*^*−/−*^ mice compared with saline-treated WT littermates (Figure 5B). Levels of both transcripts were significantly reduced in the *Mcoln1*^*−/−*^ female mice treated with CPP16-MCOLN1, indicating a reduction of glial pathology (Figure 5B). An observed reduction of *Gfap* transcripts in *Mcoln1*^*−/−*^ female mice after treatment with CPP16-MCOLN1 is consistent with our observation of reduced Gfap protein abundance using LC-MS/MS (Figure 4B). In male mice, we observed no significant reduction of either *Cd68* or *Gfap* transcripts after CPP16-MCOLN1 treatment at either high or low doses, although a trend of lower *Gfap* values with the higher dose of CPP16-MCOLN1 was apparent. These different outcomes between male vs. female groups can be potentially explained by a combination of a smaller sample size in the male group and a lower effect of CPP16-MCOLN1 treatment in male mice due to either a 3-month longer post-treatment time or sex-specific targeting of glia by CPP16 in the mouse brain that will have to be addressed in a future follow-up study.

### Pearson correlation analysis of efficacy outcomes and brain expression of *MCOLN1* transgene

**Figure 6:**
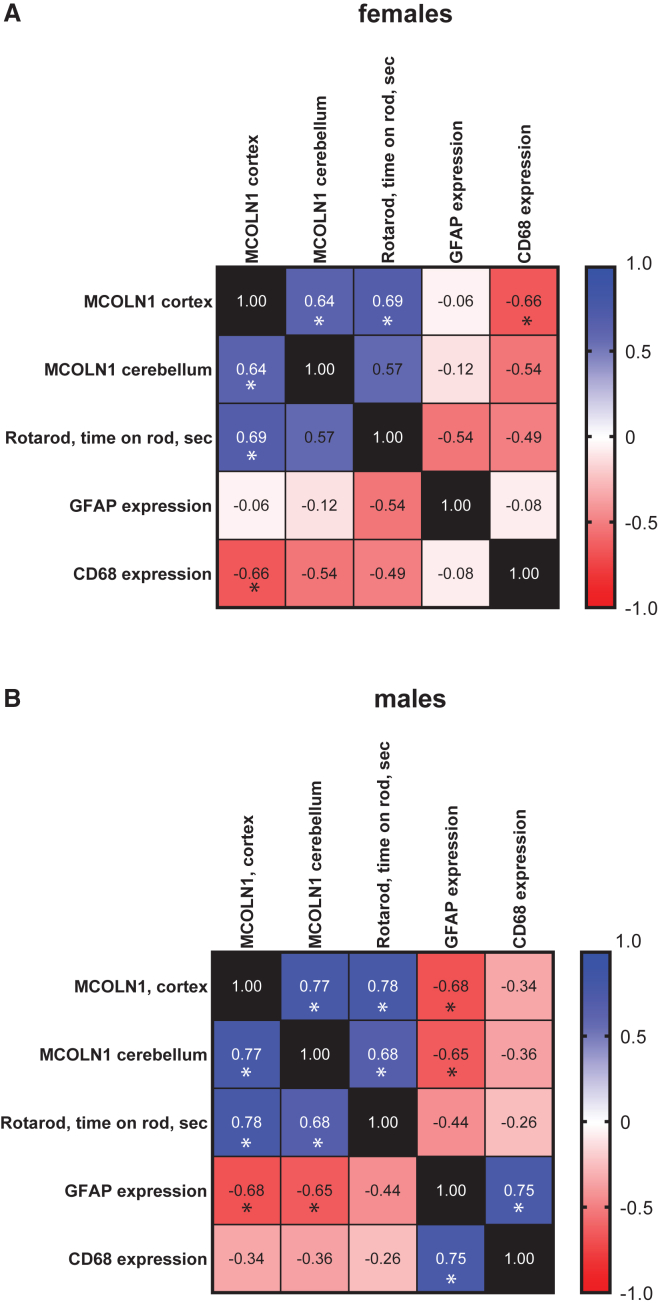
Pearson correlation analysis of efficacy outcomes and brain expression of *MCOLN1* transgene Heatmaps show the Pearson correlation coefficient between *MCOLN1* transgene expression in either the cerebral cortex or cerebellum and efficacy outcomes, including rotarod performance and expression of glial markers, Gfap, and *Cd68*. ∗*p* < 0.05. Data in the female cohort are shown in (A), and data in the male cohort are shown in (B).

Pearson correlation analysis in the male and female cohorts showed that higher *MCOLN1* transgene expression in the cerebral cortex correlated with higher expression in the cerebellum, better performance in the rotarod test and lower expression of the glial pathology markers, *Gfap* (males) and *Cd68* (females) (Figure 6), supporting the therapeutic effect of *MCOLN1* gene replacement in the *Mcoln1*^*−/−*^ mice.

## Discussion

Gene delivery to sufficient percentages of cells across broad regions in the CNS has been a major hurdle preventing the development of gene therapy for MLIV, a disease with high unmet need and critical CNS manifestations. A systemic route of administration via the bloodstream would be an ideal approach for delivering therapy to both CNS and peripheral targets. While AAV9 is the AAV serotype of broad tissue tropism and has a proven track record in human application, its CNS delivery efficiency is still suboptimal due to the limitation posed by the BBB. Extensive efforts have been made in engineering a new generation of AAV variants with different extents of BBB penetrance, such as AAV-AS, Anc80L65, AAV-PHP.B, AAV-F, 9P31, and AAV.CAP-Mac.^26^^,^^33^^,^^34^^,^^35^^,^^36^ However, it is increasingly evident that genetic drift between species poses a challenge when using AAVs selected based on small animal models for human applications. For example, a study with AAV-PHP.B, which is more than 40-fold more efficient than AAV9 in overcoming the BBB in C57BL/6 mice, does not translate into primates because of differential expression of its mouse-restricted receptor LY6A.^37^^,^^38^ We previously used AAV-PHP.B to systemically deliver a supra-physiological level of the *MCOLN1* transcript to the brain in *Mcoln1*^*−/−*^ mice and observed the correction of neurological dysfunction.^23^ The lack of translatability for AAV-PHP.B prompted us to test CPP16, the superiority of which over AAV9 translates from mice to non-human primates.^25^ CPP16 was developed through a rational-design approach by inserting a cell-penetrating-peptide (CPP)-derived peptide (“TVSALK”) into the aa588/589 site of the AAV9 capsid. Although the molecular mechanism underlying the enhanced neurotropism of CPP16 over AAV9 remains to be unveiled, increased transcytosis in the brain microvascular endothelial cells in a human BBB model and better transduction of human brain cells have been observed.^25^ Thus, CPP16 has the potential for translation into clinical testing. The fact that systemic application of CPP16-mediated *MCOLN1* gene transfer was efficacious in the mouse model, as we showed in this study, provides additional evidence supporting the further development of such therapy for MLIV patients.

The biodistribution of CPP16 and transgene expression outside of the CNS was not systemically investigated in the previous study.^25^ Our *MCOLN1* expression data after intravenous administration of CPP16-MCOLN1 in young adult mice showed broad biodistribution and expression of the transgene, not only in the brain tissue, but also in the retina, optic nerve, and other peripheral nerve tissues, as well as in other peripheral organs, such as skeletal muscle and stomach. Our previous work showed that the brain is the primary target organ for AAV-mediated *MCOLN1* gene transfer in MLIV mice.^23^ Given the complex clinical presentation of MLIV, including retinal degeneration, optic nerve pathology, and malfunctioning of parietal cells in the stomach leading to achlorhydria, a therapeutic approach involving systemic administration of CPP16-MCOLN1 that simultaneously targets the CNS, the eye, and peripheral organs may help to elicit maximal benefits in MLIV in the clinical setting. Since many genetic syndromes with early onset and CNS involvement are also characterized by peripheral pathology and visual abnormalities, including many lysosomal, mitochondrial diseases, and metabolic syndromes, CPP16 could present an attractive vector platform with applications beyond MLIV. On the other side, the risk of toxicity in vital peripheral organs, including the heart and liver, is a legitimate concern with systemic administration of a high vector dose.^39^ While we have observed no overt safety concerns in any of the CPP16-MCOLN1-treated animals, safety and toxicology outcomes were not directly assessed in this study, and a follow-up dose-finding safety/toxicology study will be required to evaluate them.

Our study was designed to assess the efficacy of the *MCOLN1* gene transfer in early symptomatic *Mcoln1*^*−/−*^ mice that have already developed neurological deficits. The timing of the intervention was selected to closely match the design for future clinical studies. Due to the early onset of the disease in humans, a vast majority of patients with MLIV have developed neurological symptoms by the time they are diagnosed and would be able to undergo treatment. MLIV families and caregivers identify early motor dysfunction as a major contributor to disability and limited quality of life in MLIV patients. Thus, a successful treatment for MLIV ought to rescue developmental motor deficits rather than delay motor deterioration. Motor deficits in *Mcoln1*^*−/−*^ mice first appear at the age of 2 months in the form of reduced rearing or vertical activity and then progress gradually to loss of ambulation due to hindlimb paralysis around 6 and premature death by 7 months of age,.^20^^,^^23^ Our observation of therapeutic benefits in the symptomatic mice after treatment with CPP16-based gene therapy suggests MLIV patients may also benefit from this approach.

Dose determination is an essential step in designing future clinical trials. We observed that both doses, 5e11 and 1e12 vg/mouse, were able to delay time of paralysis and extend lifespan of the treated *Mcoln1*^*−/−*^ mice and that only the higher dose of 1e12 vg/mouse (equivalent to approximately 5e13 vg/kg) led to significant restoration of the neurological function, improvement of the rotarod performance, full rescue of the clasping phenotype, and the overall healthy appearance. The observed dose dependence of CPP16-MCOLN1 therapy provides important guidance in selecting a range of doses for future preclinical and clinical testing. We further observed that a full therapeutic dose, 1e12 vg/mouse, of CPP16-MCOLN1 corresponds to vector biodistribution of approximately 1.7 vg/cell in the cortex. While there is no direct evidence that transgene delivery specifically to the cortex is responsible and sufficient to provide the observed benefits of motor function restoration in the MLIV model, and other brain regions transduced by CPP16 are likely contributing to the therapeutic effect in our study, cortical biodistribution may be employed as a useful readout indicative of clinical outcomes in follow-up translational dose-finding studies.

The major brain pathology hallmarks of MLIV are hypomyelination, glial activation, and enlargement of the lysosomal compartment with progressive accumulation of undigested storage material. Treatment with CPP16-MCOLN1 in young adult symptomatic mice resulted in correction of the lysosomal pathology as revealed by two independent experimental approaches, LC/MS-MS proteomics and LAMP1 immunohistochemistry, in the brain tissue of 5-month-old *Mcoln1*^*−/−*^ female mice (i.e., 3 months after treatment). We have recently reported broad upregulation of lysosomal proteins as a hallmark of pathological molecular changes in the brain of *Mcoln1*^*−/−*^ mice.^29^ A similar increase of lysosomal proteins was also reported in the single MLIV brain autopsy proteome, indicating the conservative nature of this phenomenon across species.^40^ Additionally, the upregulation of lysosomal proteins was commonly reported in other lysosomal storage disorders, such as NPC, Gaucher, mucopolysaccharidoses, and others, and is thought to represent a compensatory mechanism for impaired lysosomal function.^41^^,^^42^^,^^43^ In line with these previous findings, proteomics analysis of whole cortical tissue in this study showed similar upregulation of the lysosomal protein signature in saline-treated *Mcoln1*^*−/−*^ mice, and CPP16-mediated *MCOLN1* gene transfer resulted in the broad correction of the lysosomal protein signature, providing an additional evidence of the treatment efficacy on the molecular level.

Another major brain pathology feature in MLIV discovered by the proteomics data in the present and our previous studies was broad downregulation of the protein signature related to oligodendroglial cell lineage and myelination.^29^ It is noteworthy that early intervention via ICV administration of AAV9-MCOLN1 in neonatal *Mcoln1*^*−/−*^ in our previous work resulted in an improvement of myelination in young adult *Mcoln1*^*−/−*^ mice at 2 months. However, CPP16-mediated *MCOLN1* gene transfer in young adult symptomatic mice in the present study did not correct myelination, as shown by the lack of correction of myelination/oligodendrocyte signature in the brain proteomics dataset. The role of the *MCOLN1*-encoded lysosomal channel TRPML1 in oligodendrocyte biology has not been fully established, and the mechanism of hypomyelination in MLIV is not fully understood.^18^^,^^30^^,^^44^ We believe that the failure to rescue myelination in our studies is likely due to the time of treatment, which provides new insights into the mechanism of hypomyelination in MLIV and the therapeutic window for interventions.

It is interesting to note that while no sex-specific differences in clinical presentation have been reported in MLIV patients or MLIV mouse models (Y.G., unpublished observations), we have noticed some discrepancies in our male and female experimental cohorts in response to CPP16-MCOLN1 treatment. This includes significant rescue of the vertical activity in female but not male *Mcoln1*^*−/−*^ mice after treatment with CPP16-MCOLN1 at 1e12 vg/mouse (Figure 2). This discrepancy may be explained by suboptimal sample size and statistical power. Additionally, these data may prompt sex-specific investigations of AAV.CPP.16 biodistribution in mice and other species in future studies.

To evaluate if the phenotypical correction in the *Mcoln1*^*−/−*^-treated mice was related to *MCOLN1* expression in the cortex or cerebellum, we have performed Pearson correlation analysis in male and female cohorts (Figure 6). Our data showed a correlation of *MCOLN1* expression between cortex and cerebellum in both male and female mice. They also showed that CPP16-driven expression of *MCOLN1* in the cortex was significantly correlated with performance in rotarod test in both, male and female cohorts of mice, providing additional evidence of phenotypical rescue in the treated mice. Interestingly, despite the significant reduction of the glial activation markers in female brain tissue, we found that only microglial marker *Cd68* was significantly negatively correlated with cortical expression of *MCOLN1*. In male mice, while qPCR analysis did not show significant correction of either *Gfap* or *Cd68*, levels of *Gfap* were negatively correlated with *MCOLN1* expression in the cortex. Overall, these data further support that the elevation of *MCOLN1* expression in the *Mcoln1*^*−/−*^ mouse brain has therapeutic benefits. Follow-up dose-finding studies will be required to optimize vector dosing, biodistribution, and transgene expression for future clinical use.

In conclusion, we report a systemic AAV-based gene therapy for MLIV with the promise of translation into the clinical setting.

## Materials and methods

### Animals

*Mcoln1*^−/−^ mice were maintained as previously described.^20^ Genotyping was performed by Transnetyx using real-time qPCR (www.transnetyx.com). The *Mcoln1*^+/−^ breeders for this study were obtained by backcrossing onto a C57BL/6J background for more than 10 generations. Experimental cohorts were obtained from either *Mcoln1*^+/−^ × *Mcoln1*^+/−^ or *Mcoln1*^+/−^ × *Mcoln1*^−/−^ mating. *Mcoln1*^+/−^ and *Mcoln1*^+/+^ littermates were used as controls. Experiments were performed according to the Institutional and National Institutes of Health guidelines and approved by the Massachusetts General Hospital Institutional Animal Care and Use Committee. Animals were assigned to the experimental groups in a random order. Handling and testing were performed by investigators blinded to treatment.

### Virus preparation and titration

The previously described expression plasmid pAAVsc-JeT-MCOLN1-pA was used to generate scAAV-CPP16-MCOLN1 used in this study.^23^ AAV vector production was done as previously described.^25^ Briefly, HEK293T cells were co-transfected by three plasmids: a pAAV-RC, a pHelper (240071–12, Agilent Technologies), and an ITR-flanked AAV plasmid (pAAVsc-JeT-MCOLN1-pA) using polyethyleneimine (Cat. no 23966, Polysciences). AAV was collected from serum-free culture media at 72 h and from the cells and media 120 h post transfection. Viral particles were purified by iodixanol gradient (15%, 25%, 40%, and 60%) ultracentrifugation, then concentrated and desalted using 100K Millipore Amicon filter unit (UFC910008, 100 K MWCO) and formulated in Dulbecco’s PBS. Any viral particle precipitation was re-suspended before application.

AAV titers were determined using quantitative PCR. Briefly, AAV samples were treated with DNase I to remove contaminating DNA, followed by sodium hydroxide treatment to lyse the AAV capsid and release the scDNA. pAAVsc-JeT-MCOLN1-pA plasmid was digested with EcoRI (NEB) to recover the ITR-flanked AAV genome to be used as standard. Quantitative PCR was performed with primers targeting the *MCOLN1* transgene: forward primer 5′-CAGCACGGAGACAACAGCTT and reverse primer 5′- CAGGGAGCAGGTGAGGATGA.

### Intravenous injections

Mice were restrained in a plexiglass container, and the tail veins were dilated under a heat lamp for 1 min. We used 30G needle insulin syringes (cat. No 328466, BD, San Diego, CA) to inject 150–200 μL either 0.9% saline (Hanna’s Pharmaceutical Supply) or 5e11 vg or 1e12 vg scAAV-CPP.16-JeT-MCOLN1 via the tail veins of each mouse.

### Behavioral testing

Open field testing was performed at four months of age under regular light conditions. Testing was done at the same time each day to minimize the effects of the circadian cycle, and males were tested the day before females. Each mouse was placed in the center of a 27 × 27 cm^2^ Plexiglass arena, and the horizontal and vertical activities were recorded by the Activity Monitor program (Med Associates). Data were analyzed during the first 15 min in the arena.

Motor coordination and balance were tested on an accelerating rotarod (Ugo Basile). Latency to fall from the rotating rod was recorded in three trials on one day (accelerating speed from 4 to 40 rpm over 5 min) following a training day of two trials. Animals were tested monthly, starting from 4 months of age to 8 months of age or until they reached the euthanasia endpoint, such as complete paralysis.

To visualize the clasping reflex in the mice, one investigator held each mouse by the tail while another took a 2-s-long video. Screengrabs of the videos are displayed in Figure 2. To determine the euthanasia time point for the knockout mice, the righting reflex of each mouse was evaluated every 2–3 days starting at 5.5 months of age. When mice could no longer right themselves in fewer than 10 s after being placed on their backs, they were euthanized.

### OCT

*In vivo* imaging of the retina via OCT was performed as described previously.^45^ Mice were anesthetized in a mobile isoflurane induction chamber with 2% of isoflurane at 2 L/min O_2_. Pupils of the mice were dilated using 2.5% phenylephrine and 1% tropicamide. About 0.5% of the proparacaine was used as a topical anesthetic. SD-OCT imaging was performed using a Leica EnvisuR2210 (Biooptigen). Measurements were made 500 μm from the optic nerve for the central retina and 1.5 mm from the optic nerve for the peripheral retina. Linear B-scans of central and peripheral retina were performed, and the thickness of the total retinal, and retinal pigment epithelium. Outer segments and outer nuclear layers were measured using Bioptigen Diver software (Bioptigen, Inc.) using automated segmentation. Each OCT image comprises 100 B-scans, with each B-scan consisting of 1000 A-scans. For quantification of retinal thickness, two representative images per eye were analyzed for four images per mouse.

### Tissue collection and processing

Mice were sacrificed using a carbon dioxide chamber. Immediately after euthanasia, mice were transcardially perfused with ice-cold PBS. The brain was removed and bisected down the midline; one-half was post-fixed in 4% paraformaldehyde (Electron Microscopy Sciences) in PBS for 48 h, washed with PBS, cryoprotected in 30% sucrose in PBS for 24 h, snap-frozen in isopentane, and stored at −80°C. The other one-half was further dissected and, along with all other tissues, snap frozen on dry ice.

### RNA extraction and qPCR analysis

Mouse tissues were either homogenized using QIAzol lysis reagent (Qiagen) in a Tissue Lyser instrument (Qiagen) or with a mortar and pestle in buffer RLT (Qiagen) using a 19G needle and syringe. Total RNA isolation from homogenized tissues was performed using Qiagen RNeasy kit (Qiagen) and genomic DNA was eliminated by performing DNase (Qiagen) digestion on columns following the provider’s protocol. cDNA was produced from 500 ng of starting RNA using High-Capacity cDNA Reverse Transcription kit (Applied Biosystems). After dilution, 40 ng of the cDNA was used for qPCR using LightCycler 480 Probes Master mix (Roche Diagnostics) or TaqMan gene expression assays (see details below) on the LightCycler 480 (Roche Diagnostics). Human tissue was obtained from the NIH NeuroBioBank’s Brain and Tissue repository at the University of Maryland, Baltimore.

To perform relative gene quantification, TaqMan probes (Applied Biosystems) were used to measure mouse *Mbp* (FAM)-Mm01266402_m1, mouse *Gfap* (FAM)-Mm01253033_m1, mouse *Cd68* (FAM)-Mm03047343_m1, mouse *Lamp1* (FAM)-Mm00495262_m1, and human mucolipin-1 (FAM)-Hs01100653_m1, using mouse GAPDH (FAM)-Mm99999915_g1 as reference. The ΔΔCt method was used to calculate relative gene expression, where Ct corresponds with the cycle threshold. ΔCt values were calculated as the difference between Ct values from the target gene and the housekeeping gene GAPDH.

To perform absolute RNA quantification, three TaqMan probes (APZTMGC) were designed to measure AAV-JeT-driven expression of *MCOLN1* transgene: forward 5′-GGTCGCGGTTCTTGTTTGT-3′, reverse 5′- GAAGCCGCTCGGTCTCT-3′, probe 5′- CCCTGTGATCGTCACTTGACAGTGT-3’. To obtain a standard curve, the scAAV-JeT-MCOLN1 plasmid was linearized with HindIII (New England Biolabs) as directed by the manufacturer. The DNA concentration was determined after digestion, and the number of copies of DNA/μL was calculated assuming a molar mass of 650 g/mol per base pair and a fragment length of 5,158 nt. A standard curve with serial dilutions of the linearized plasmid ranging from 4 × 10^2^ to 4 × 10^7^ copies per reaction was used to determine the absolute number of cDNA transcripts. For a comparative qRT-PCR *MCOLN1* expression assay in human and mouse cortical tissue, a TaqMan probe set (FAM)-Hs01100653_m1 (Applied Biosystems) was used.

### Immunohistochemistry, imaging, and analysis

We cut 40-μm coronal brain sections using a cryostat (Leica Microsystems) and collected into 96-well plates containing cryoprotectant (30% ethylene glycol, 15% sucrose in TBS). Staining of free-floating sections was done in a 96-well plate, and samples were randomized and coded to create blinded conditions for analysis. Sections were blocked in 5% normal goat serum (NGS), 2% bovine serum albumin (BSA), 1% glycine, and 0.1% Triton X-100 in PBS. The primary antibodies against LAMP1 (Rat 1:1000, BD, Cat ID: 553792) and Gfap (Mouse, 1:1000; Cell Signaling Technology, Cat. ID: 3670S) were diluted in 5% NGS and 2% BSA and applied overnight at 4°C. The next day, sections were incubated in secondary antibodies goat-anti-rat Alexa Fluor 633 (1:500; Invitrogen) or goat-anti-mouse Alexa Fluor 555 (1:500; Invitrogen) in 1% NGS for 1–2 h at room temperature, and mounted onto glass SuperFrost plus slides (Fisher Scientific) with Immu-Mount (Fisher Scientific). Images were acquired using DM8i Leica Inverted Epifluorescence Microscope with Adaptive Focus (Leica Microsystems), Hamamatsu Flash 4.0 camera, and advanced acquisition software package MetaMorph 4.2 (Molecular Devices, LLC) with an automated stitching function. The exposure time was kept the same for all sections within the same immunohistochemistry experiment. Image analysis was performed using Fiji software (NIH). Particle analysis and percentage-of-area measurements for Lamp1 and Gfap images were made after the same thresholding settings were applied to all images. The image analysis was done by an investigator blinded to the genotype and treatment groups. All images were decoded after the measurements were taken. Area and particle size values were averaged per genotype/treatment group and compared between groups using ordinary one-way ANOVA test and Dunnett’s correction for multiple comparisons in the GraphPad Prizm v9 software.

### MS proteomic analysis

Protein extraction from the cerebral cortex, TMT labeling, and LC-MS/MS analysis were performed as previously described.^29^ Raw data were submitted for analysis in Proteome Discoverer 3.0.1.23 (Thermo Fisher Scientific) software with Chimerys (MSAID). Assignment of MS/MS spectra was performed using the Sequest HT algorithm and Chimerys by searching the data against a protein sequence database, including all entries from the Mouse Uniprot database (SwissProt 19,768 2019) and other known contaminants such as human keratins and common lab contaminants. Sequest HT searches were performed using a 20 ppm precursor ion tolerance and requiring each peptide’s N-/C termini to adhere with trypsin protease specificity while allowing up to two missed cleavages. The 18-plex TMT tags on peptide N termini and lysine residues (+304.207146 Da) were set as static modifications, and carbamidomethyl on cysteine amino acids (+57.021464 Da), while methionine oxidation (+15.99492 Da) was set as variable modification. An MS2 spectra assignment false discovery rate of 1% on the protein level was achieved by applying the target decoy database search. Filtering was performed using a Percolator (64-bit version, Acierno et al^1^). For quantification, a 0.02 m/z window centered on the theoretical m/z value of each of the six reporter ions, and the intensity of the signal closest to the theoretical m/z value was recorded. Reporter ion intensities were exported in the result file of the Proteome Discoverer 3.0 search engine as an Xcel table. The total signal intensity across all peptides quantified was summed for each TMT channel, and all intensity values were adjusted to account for potentially uneven TMT labeling and/or sample handling variance for each labeled channel.

PSM-level data from each TMT channel was analyzed in R. The initial preprocessing involved missing value imputation using random forest and the removal of PSMs with incomplete or multiple protein mapping or isolation interference of more than 70. The preprocessed data were aggregated to the protein level and normalized via Variance Stabilizing Normalization. Differential expression analysis was performed using the R package limma to compare proteomic profiles of untreated *Mcoln1*^*−/−*^ cortical samples with WT and AAV-MCOLN1-treated *Mcoln1*^*−/−*^ mice. Visualization was done through volcano plots, highlighting proteins with a significant difference (p < 0.05) and a log fold-change of more than |0.5|. Significantly altered proteins in the *Mcoln1*^*−/−*^ vs. WT comparison were utilized to create a heatmap showing abundance differences across all samples in three experimental groups.

## Data and code availability

Raw data for this study are available from the corresponding author upon reasonable request.

## Supplemental information

Document S1. Figures S1‒S3

Table S1. Protein abundances in whole cortical homogenates in *Mcoln1*^-/-^ -saline, *Mcoln1*^-/-^ -AAVCPP16-*MCOLN1* and WT-saline mice

Table S2. Common contaminants excluded from the analysis

Table S3. UP and DOWN regulated proteins in whole cerebral cortex homogenates from WT and *Mcoln1*^-/-^saline-treated mice

Table S4. UP and DOWN regulated proteins in whole cerebral cortex homogenates from *Mcoln1*^-/-^ - CPP16-*MCOLN1* and *Mcoln1*^-/-^ -saline mice

Document S2. Article plus supplemental information
