# Supplementary material for: A blood-brain barrier-penetrant AAV gene therapy improves neurological function in symptomatic mucolipidosis IV mice
Source: Mol Ther Methods Clin Dev. 2024 May 21;32(2):101269. doi: 10.1016/j.omtm.2024.101269 (PMC11201152; doi:10.1016/j.omtm.2024.101269)
Supplement: Document S1. Figures S1‒S3 [file mmc1.pdf]

OMTM, Volume *32*

Supplemental information

A blood-brain barrier-penetrant AAV gene therapy improves neurological function in symptomatic mucolipidosis IV mice

Madison L. Sangster, Martha M. Bishop, Yizheng Yao, Jessica F. Feitor, Sanjid Shahriar, Maxwell E. Miller, Anil K. Chekuri, Bogdan Budnik, Fengfeng Bei, and Yulia Grishchuk

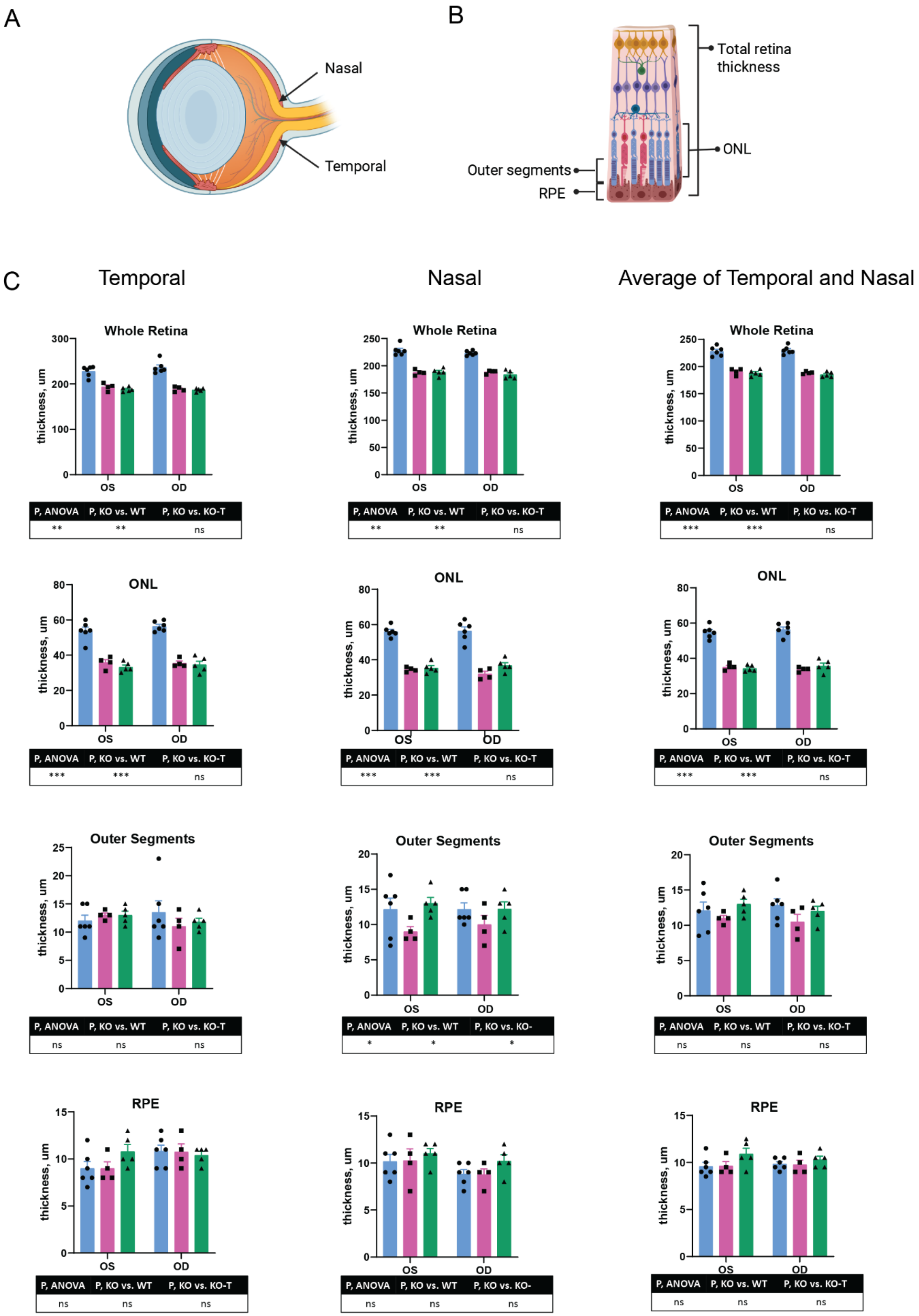
A
Nasal
Temporal
B
Total retina thickness
ONL
Outer segments
RPE
C
Temporal
Nasal
Average of Temporal and Nasal
Whole Retina
ONL
Outer Segments
RPE
thickness, um
OS
OD
P, ANOVA
P, KO vs. WT
P, KO vs. KO-T
ns

Figure S1.

Figure S1. Systemic administration of CPP16-*MCOLN1* in young adult symptomatic *Mcoln1*$^{-/-}$ mice did not improve retinal thickness. A. Schematic representation of the mouse eye, showing positions “nasal” and “temporal” where retinal measurements took place. **B.** Schematic presentation of the retinal structure showing acquired retinal measurements. **C.** Retinal layer thickness in WT- saline (blue), *Mcoln1*$^{-/-}$ - saline (pink) and *Mcoln1*$^{-/-}$- CPP16-*MCOLN1* (green) temporal and nasal sections of retina in the left (OS) and right (OD) eyes. Individual, group mean, and SEM values are presented, n=6 (WT saline), n=4 (KO saline), n=5 (KO CPP16-*MCOLN1*). Statistical analysis was done using one-way ordinary ANOVA and multiple comparison test using GraphPad Prizm v.9.

Figure S2.

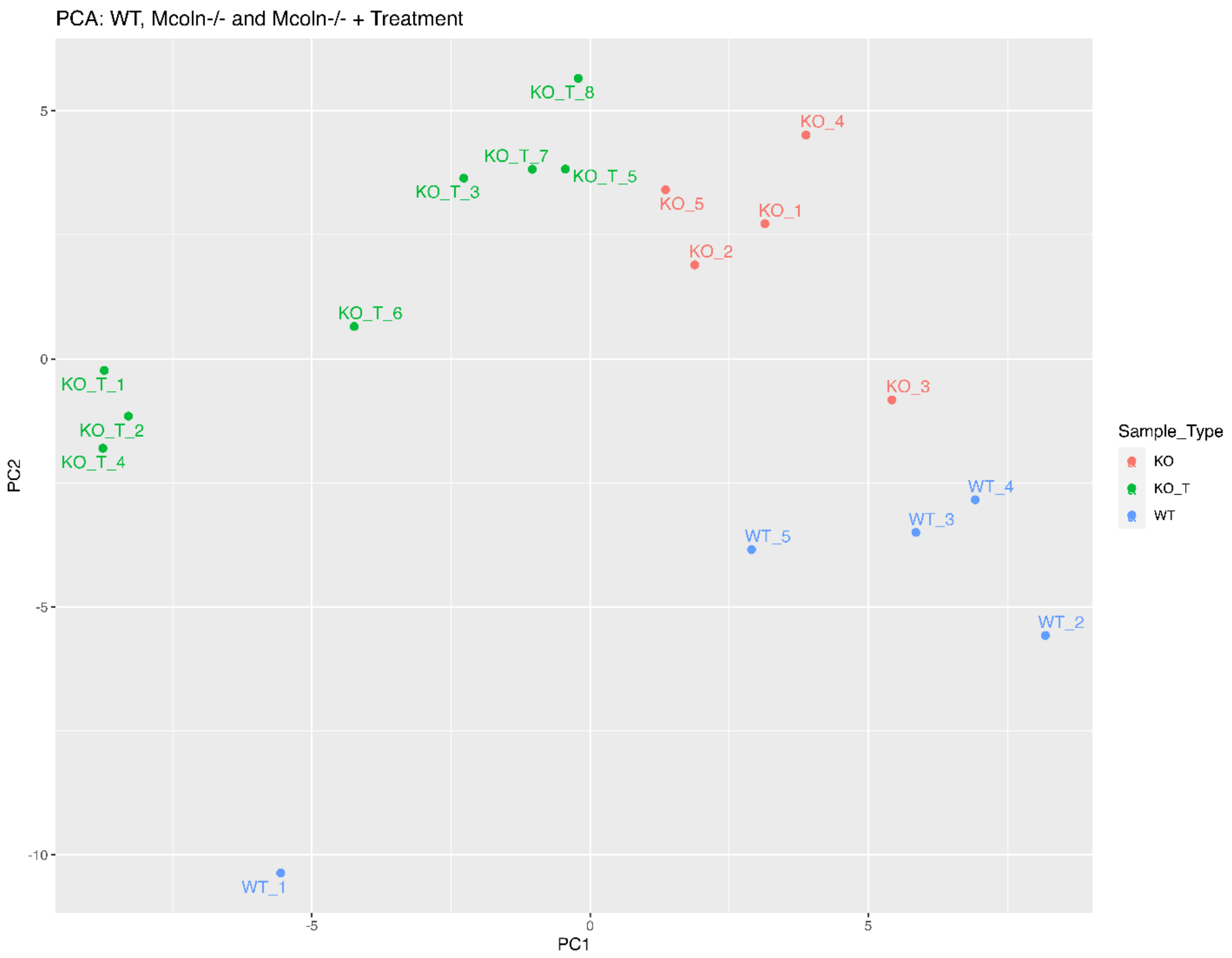

Figure S2. Principal component analysis (PCA) showing separation of *Mcoln1*-/- -saline (KO), *Mcoln1*-/- -CPP16-*MCOLN1* (KO-T) and WT-saline (WT) whole cortical homogenate samples via LC-MS/MS.

Figure S3.

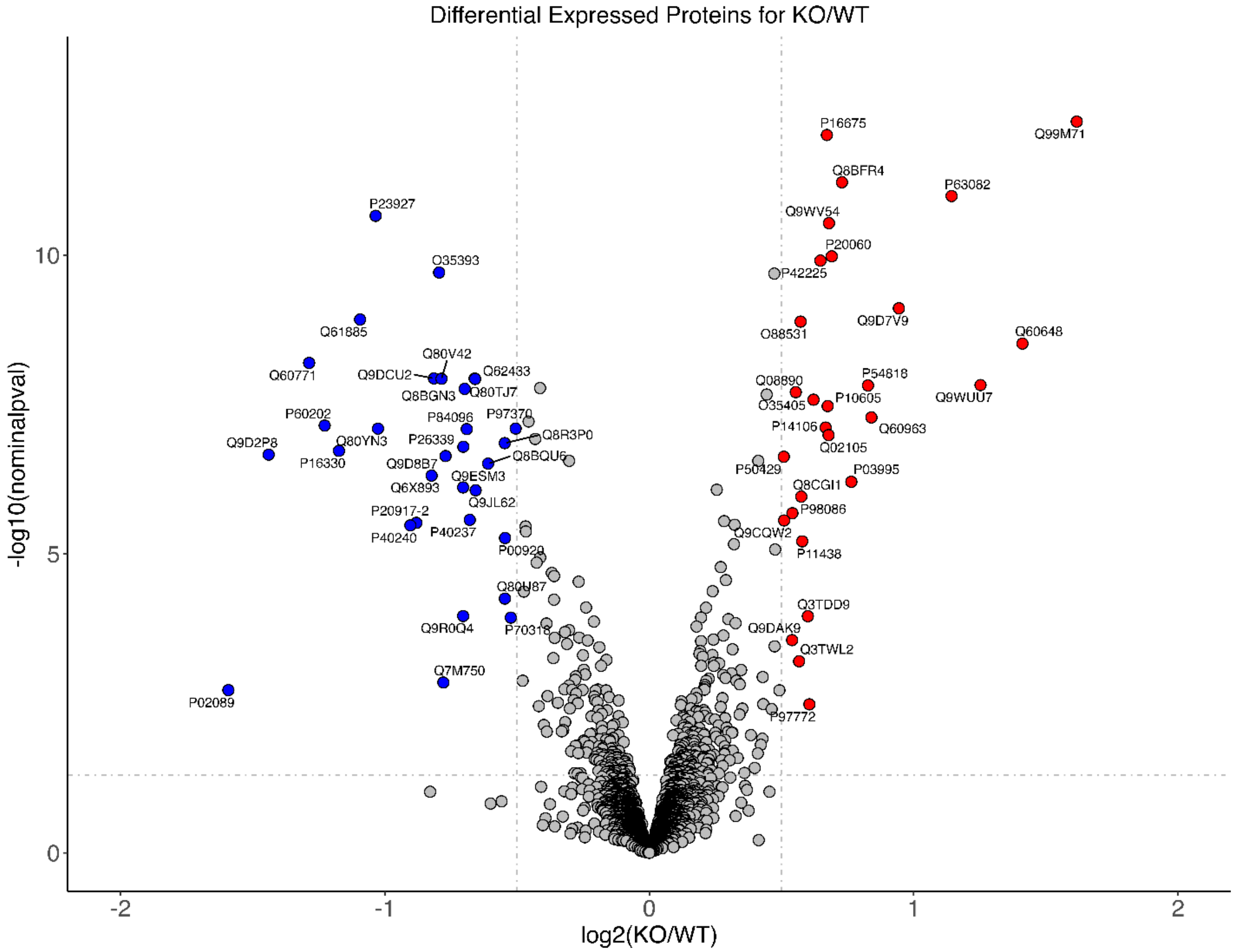

Figure S3. Volcano plot showing upregulated and downregulated proteins in whole cortical homogenates from *Mcoln1*-/- and WT mice; log10 p<1.3 (p<0.05), log2FC>0.5.

Table S1. Protein abundances in whole cortical homogenates in *Mcoln1*-/- -saline, *Mcoln1*-/- -AAV-CPP16-*MCOLN1* and WT-saline mice.

Table S2. Common contaminants excluded from the analysis.

Table S3. UP and DOWN regulated proteins in whole cerebral cortex homogenates from WT and *Mcoln1*-/- saline-treated mice.

Table S4. UP and DOWN regulated proteins in whole cerebral cortex homogenates from *Mcoln1*$^{-/-}$ -CPP16-*MCOLN1* and *Mcoln1*$^{-/-}$ -saline mice.
